# Insusceptibility to disinfectants in bacteria from animals, food and humans—is there a link to antimicrobial resistance?

**DOI:** 10.3389/fmicb.2014.00088

**Published:** 2014-03-18

**Authors:** Karin Schwaiger, Katrin S. Harms, Meike Bischoff, Petra Preikschat, Gabriele Mölle, Ilse Bauer-Unkauf, Solveig Lindorfer, Sandra Thalhammer, Johann Bauer, Christina S. Hölzel

**Affiliations:** ^1^Animal Hygiene, Wissenschaftszentrum Weihenstephan für Ernährung, Landnutzung und Umwelt, Technische Universität MünchenFreising, Germany; ^2^Bavarian Health and Food Safety Authority (LGL)Erlangen, Germany

**Keywords:** disinfectant, biocide, antiseptic, antimicrobial resistance, QAC, formic acid, enterococci, aminoglycoside

## Abstract

*Enterococcus faecalis* (*n* = 834) and *Enterococcus faecium* (*n* = 135) from blood and feces of hospitalized humans, from feces of outpatients and livestock and from food were screened for their susceptibility to a quaternary ammonium compound (didecyldimethylammoniumchloride, DDAC) and to 28 antibiotics by micro-/macrodilution. The maximum DDAC-MIC in our field study was 3.5 mg/l, but after adaptation in the laboratory, MIC values of 21.9 mg/l were observed. Strains for which DDAC had MICs > 1.4 mg/l (“non-wildtype,” in total: 46 of 969 isolates/4.7%) were most often found in milk and dairy products (14.6%), while their prevalence in livestock was generally low (0–4%). Of human isolates, 2.9–6.8% had a “non-wildtype” phenotype. An association between reduced susceptibility to DDAC, high-level-aminoglycoside resistance and aminopenicillin resistance was seen in *E. faecium* (*p* < 0.05). No indications for a common source of non-wildtype strains were found by RAPD-PCR; however, several non-wildtype *E. faecalis* shared the same variant of the *eme*A-gene. In addition, bacteria (*n* = 42) of different genera were isolated from formic acid based boot bath disinfectant (20 ml of 55% formic acid/l). The MICs of this disinfectant exceeded the wildtype MICs up to 20-fold (staphylococci), but were still one to three orders of magnitude below the used concentration of the disinfectant (i. e., 1.1% formic acid). In conclusion, the bacterial susceptibility to disinfectants still seems to be high. Thus, the proper use of disinfectants in livestock surroundings along with a good hygiene praxis should still be highly encouraged. Hints to a link between antibiotic resistance and reduced susceptibility for disinfectants—as seen for *E. faecium*—should be substantiated in further studies and might be an additional reason to confine the use of antibiotics.

## Introduction

Prevention of zoonoses—including the spread of antimicrobial resistant bacteria—is mainly a question of how to reduce the prevalence of contagious microorganisms. Whenever actions are taken to reduce the spread of bacteria in livestock, effective disinfection is crucial.

Antimicrobial resistance is basically increasing whenever resistant bacteria are selected by antimicrobial use (Bronzwaer et al., 2002; Lipsitch and Samore, 2002; Livermore, 2005). A certain antibiotic agent might directly select for resistance directed against itself, might indirectly (cross-)select for cross-resistance to chemically related agents and/or might (co-)select for co-resistance to unrelated substances (Shah, 2005), the latter based e.g., on co-transfer of resistance genes due to genetic linkage on mobile elements. Besides co-selection posed by antibiotic agents, diverse other co-selectors are discussed, e.g., heavy metal ions (Berg et al., 2005; Baker-Austin et al., 2006; Hölzel et al., 2012), pesticides (Bordas et al., 1997), or disinfectants (Levy, 2000). For several bacterial species, including methicillin resistant *Staphylococcus aureus*, a link between resistance against antibiotics and reduced susceptibility for disinfectants has been described in the past (Heir et al., 1999; Bjorland et al., 2001, 2005; Sidhu et al., 2002a). At the same time, other studies did not find clear indications for co-resistance against antibiotics and disinfectants (Suller and Russell, 2000; Loughlin et al., 2002). Enterococci—apart from VRE—have rarely been investigated for this correlation up to now, despite the fact that enterococci are emerging—meanwhile maybe better called: “emerged”—nosocomial pathogens (Tailor et al., 1993; Willems and van Schaik, 2009) with a high recombination potential (Aarestrup et al., 2002; Leavis et al., 2006; Palmer and Gilmore, 2010; Schwaiger et al., 2011, 2012).

Acquired insusceptibility to disinfectants can be conferred by newly acquired genes (like *qac*-genes, Poole, 2002) or by mutations, e.g., of intrinsic multidrug transporters (like *nor*A, *eme*A, Kaatz et al., 1993; Ng et al., 1994; Jonas et al., 2001) or of target structures (e.g., cell membranes), although the latter is described to be a rare event with biocides (Poole, 2002). To slightly complicate the situation, the mere presence of *qac*-genes seems to correlate only weekly with phenotypic insusceptibility; instead, induced overexpression of *qac*-genes might explain a reduced phenotypic susceptibility in a more satisfactory way (Cervinkova et al., 2012).

Bacteria that are contemporaneously resistant to antibiotics and disinfectants are a worrying scenario. According to their telling name, multidrug transporters have more than one substrate (which might be chemically unrelated to each other). In this way, multidrug transporters seem to be predisposed to confer multiresistance. However, in most cases the unspecific efflux provided by multidrug transporters leads to not more than slightly elevated MIC-values below clinical antimicrobial resistance (Lee et al., 2003). Multiresistance might also result from a combination of newly acquired genes. Such genes can be physically linked on common genetic elements like plasmids or transposons and might spread to other strains, species or genera, independent from their original carriers—as shown for *qac*-genes and *bla*-genes (Bjorland et al., 2005). Moreover, links between antibiotic resistance and tolerance to disinfectants might also be caused by the clonal spread of co-resistant strains—irrespective of whether this co-resistance is encoded within the intrinsic or within the variable gene pool. This might be the reason why most evidence for co-resistance to antibiotics and disinfectants is found in MRSA-strains, which are known to spread often in clonal complexes (Oliveira et al., 2002). Although antibiotic resistance in enterococci is thought to spread mainly via the horizontal spread of resistance genes (Willems et al., 2011), successful clonal lineages have also been identified (Nallapareddy et al., 2005; Leavis et al., 2006).

In case of co- or cross-resistance to disinfectants, any discussion should be very carefully balanced, since adequate disinfection is crucial for any hygienic concept (Cozad and Jones, 2003), in hospitals as well as on farms or in the food producing industry. Russell (2000) emphasizes that the term cross-*resistance* is problematic for the situation seen up to now with antibiotics and disinfectants, since all studies which report on this phenomenon use disinfectant concentrations far below practically applied concentrations. This means that, up to now, we are talking about “microbiological” or “epidemiological” resistance to disinfectants. Such strains differ from the wildtype by elevated MIC-values, but still have MIC-values below “clinically” or “in praxi” used (breakpoint) concentrations.

The present study aimed to investigate a representative number of *Enterococcus faecalis* (*n* = 824) and *Enterococcus faecium* (*n* = 130) from diverse sources (hospital/food industry/farm animals) for their susceptibility to a disinfectant (didecydimethylammoniumchlorid, DDAC) and 22 antibiotic agents. The distribution of strains with DDAC-MICs > or < 1.4 mg/l within antibiotic resistant and susceptible strains was assessed. In case of elevated DDAC-MIC-values strains were investigated for clonal relationship using amplicon-typing (RAPD-PCR). In several isolates, the *eme*A-genotype, coding for a multidrug efflux transporter, was further investigated by melting curve analysis and sequencing.

The higher the initial MIC of strains the more rapidly they might be adapted to rising concentrations of disinfectants, as shown by Sidhu et al. (2002b). Therefore, we performed additionally an exploratory adaptation test with the DSM 2570 reference strain and three strains with DDAC_MICs > 1.4 mg/l.

To have a first glance on real life conditions, different bacterial species were additionally isolated from the disinfectant fluid of boot baths and tested for their MIC-values for DDAC, formic acid and antibiotics.

## Materials and methods

### Collection and identification of strains

All *Enterococcus* strains (*n* = 954) were collected and identified in the frame of the second Bavarian antimicrobial resistance monitoring project (BAVMAP II, Bischoff et al., 2012) by species-specific PCR as previously described (Bischoff et al., 2012). Isolates from disinfectant boot bath were taken in three different ways:

- By direct culturing from the disinfectant fluid.
- By means of a sterile swab which was subsequently introduced in TS-broth (30 g cold filterable tryptone soya broth per 1 l of distilled water) supplemented with a specific inactivation medium for organic acids. The inactivation medium was recommended by the German Veterinary Medical Society (DVG, 2008) and was added to the disinfectant with final concentrations of 3% tween 80, 3% saponin, 0.3% lecithin, 0.3% sodium thiosulphate, 0.1% histidin, 0.01 mol/l disodium hydrogenphosphate.
- By means of a sterile swab as described above, but without inactivation medium.
  Swabs and disinfectant fluid were streaked on nutrient agar with 7% sheep blood, on Gassner agar, CATC-agar, and Schaedler agar; plates were incubated aerobically or anaerobically (Schaedler Agar) for 48 h. Single colonies were picked, subcultured, and preliminary identified by colony morphology, Gram staining, oxidase-, catalase-, and indole-reaction, as well as growth and characteristics on selective agar (Fluorocult agar, VWR, Darmstadt, Germany; CATC agar, VWR; Baird Parker agar, Otto Nordwald, Hamburg, Germany). Results were confirmed by commercial biochemical test sets (API®/ID-32, bioMérieux, Nürtingen, Germany; BBL™ Crystal™ Enteric/Nonfermenter ID Kit, BD, Heidelberg, Germany) and species-specific PCR (enterococci, Bischoff et al., 2012).

### Susceptibility tests

#### Disinfectants

***Microdilution***. The susceptibility of all 969 enterococcal strains to didecyldimethylammoniumchloride (DDAC) (Tradename Sokrena, Bode Chemie, Hamburg, Germany) was assessed in a microdilution procedure as previously described (Bischoff et al., 2012). In brief, TS-Broth (30 g cold filterable Tryptone Soya Broth per 1 l distilled water) containing 1 × 10^8^–1 × 10^9^ cfu of the test strain per ml was diluted 1:100 in water of standardized hardness (WSH, consisting of 0.89 g waterfree NaCl_2_ and 0.5 g MgCl_2_ × 7 H_2_O per 3.3:l of distilled water), resulting in bacterial concentrations of 1 × 10^6^–1 × 10^7^ cfu per ml. Then, 100 μl of a double concentrated DDAC–WSH solution was manually placed into each well of a 96 well microtiter plate. Thirteen ml of TS-broth was inoculated with 226 μl of the 1:100 diluted bacterial suspension, and 100 μl of the resulting suspension was pipetted into each (DDAC-WSH filled) well of the microtiter plate by a semiautomatic dispenser (Micronaut Sprint, Genzyme-Virotech). Microtiter plates were covered with transparent plastic films and incubated at 37°C for 72 h, as specified in the instructions of the German Veterinary Medical Society (DVG, 2008); the microbial growth was visually investigated after 24 and 72 h.

***Macrodilution***. MIC-values > 1.4 mg/l in the microdilution test were confirmed in the DVG-reference macrodilution test. Bacterial suspensions were prepared as for the microdilution test; 100 μl of these suspensions were added to 4.9 ml of TS-broth + DDAC (DDAC-concentrations of 0.14–1400 mg/l, corresponding to a ready-to-use solution with 0.0002–2% = 0.002–20 ml disinfectant stock solution per liter) and incubated for 72 h. (evaluation after 24 and 72 h) at 37°C.

The lowest concentrated ungrown (clear) test-tube was noted as minimum inhibitory concentration; from this tube, 0.1 ml was plated on blood agar after the 72 h of incubation in order to determine preliminarily whether MIC-values corresponded to minimum bactericidal (MBC)-values.

***Classification***. According to Bischoff et al. (2012), isolates were classified as “non-wildtype” if DDAC had MICs > 1.4 mg/l. This classification was additionally supported by the data of 27 enterococcal isolates from wild Finish grouse, with a range of 0.35–1.4 mg/l and a median of 0.35 mg/l (data not shown).

#### Antibiotic agents

Antibiotic resistance was determined by microdilution following DIN 58940-81 recommendations as previously described (Hölzel et al., 2010). All 969 enterococci were tested for antibiotic resistance using a panel of 22 agents. *Enterobacteriaceae* from boot bath disinfectant were tested with 13 and Gram-positives with 20 antibiotics (Table 1).

**Table 1:** **Antibiotic agents, concentration ranges, and breakpoints**.

**Name of antibiotic**	**Range (mg/l)**	**Breakpoint (mg/l)**		**Tested against**	**References**
		**S≤**	**R >**		
Amoxicillin/ Clavulanic acid	1/2–128/2 0.125/2–8/2	8 4	8 8	Gram-neg. Gram-pos.	EUCAST
Ampicillin	1/2–128/2 0.125/2–8/2	8 4	8 8	Gram-neg. Gram-pos.	EUCAST
Cefaclor	1–8	1	4	Gram-neg.	DIN^1^
Cefazolin	0.125–16	4	8	Gram-neg. Gram-pos.	DIN
Cefoxitin	2–16	4	8	Gram-neg.	DIN
Cefuroxime	0.5–64 1–8	8 4	8 8	Gram-neg. Gram-pos.	EUCAST DIN
Chloramphenicol	2–64	16	16	Enterococci	DANMAP^a^
Ciprofloxacin	0.0625–8 0.25–32	0.5 1	1 2	Gram-neg. Gram-pos.	EUCAST DIN
Clindamycin	0.0625–8	1	4	Gram-pos.^b^	DIN
Doxycycline	0.5–4 0.125–16	1	4	Gram-neg. Gram-pos.	DIN
Enrofloxacin	0.0625–8	0.25	2	Gram-neg. Gram-pos.	CLSI
Erythromycin	0.0625–8	1	4	Gram-pos.	DIN
Florfenicol	2–64 2–32	16	16	Gram-neg. Gram-pos.	DIN
Fosfomycin	8–64	32	32	Gram-pos.	EUCAST^c^
Gentamicin	0.25–32	2 1	4 1	Gram-neg. Gram-pos.	EUCAST EUCAST^c^
Gentamicin HL^d^	512	512	512	Enterococci	DANMAP
Imipenem	0.125–16	2 4	8 8	Gram-neg. Gram-pos.	EUCAST
Linezolid	0.125–16	4	4	Gram-pos.	EUCAST
Meropenem	0.125–16	2	8	Gram-neg.	EUCAST
Mezlocillin	2–256	4	16	Gram-pos.	DIN
Moxifloxacin	0.0625–8	1	2	Gram-pos.	DIN
Rifampicin	0.5–4	0.006	0.5	Gram-pos.	EUCAST^b^
Streptomycin HL^d^	256–2048	512	512	Enterococci	EUCAST
Synercid	0.125–16	1	4	Gram-pos.^a^	EUCAST
Teicoplanin	0.25–32	2	2	Gram-pos.	EUCAST
Tobramycin	0.25–32	2	4	Gram-neg.	EUCAST
Tylosin	0.5–4	4	4	Gram-pos.	DANMAP
Vancomycin	0.5–64	4	4	Gram-pos.	EUCAST

a *DIN: DIN 58940-4; DANMAP: all DANMAP 2004, except tylosin: DANMAP 1997 (http://www.danmap.org/)*.

b *Except E. faecalis*.

c *For staphylococci*.

d *High level concentrations indicative for aminoglycoside/penicillin synergism*.

### Adaptation to increasing DDAC-concentrations

Four isolates of *E. faecalis* (*E. faecalis* DSM 2570 and 3 field strains with non-wildtype MIC-values for DDAC of 2.7 mg/l) were adapted to grow in gradually increasing DDAC concentrations for up to 70 days. Several colonies of the isolates were picked from blood agar plates and were cultured in 10 ml of TS-broth (37°C on a shaker at 200 rpm). After 24 h, 1 ml suspension was transferred into 9 ml of TS-broth supplemented with DDAC (0.7–21.9 mg/l = 0.001–0.03%; starting at log 2_MIC_ - 1) and incubated as mentioned above for another 24 h. The adaption process was initiated as follows: for each strain, the highest concentrated DDAC suspension which still allowed growth (c_max_) was centrifuged. The resulting pellet was washed thrice with WSH and homogenized. One ml of this WSH suspension was transferred into TS-broth containing DDAC in the following final concentrations (i) log 2 c_max_ − 1 (ii) log 2 c_max_ and (iii) log 2 c_max_ + 1 and incubated as mentioned above. Accompanying, one loop-full of the inoculating suspension was streaked onto sheep-blood-agar in order to ensure the purity of cultures. This process was repeated every 24 h.

### DNA-extraction

DNA was extracted from pure bacterial cultures of *E. faecalis*, *E. faecium*, or *E. coli* using cetyltrimethylammoniumbromide (CTAB) as previously described (Korthals et al., 2008)

### RAPD-PCR

In order to investigate whether DDAC-tolerant strains were clonally related, isolates were amplicon-typed by RAPD-PCR.

#### E. faecalis

Amplicons of *E. faecalis* were generated using primer M13R2: GGAAACAGCTATGACCATGA (Martin et al., 2005). PCR-conditions were denaturation at 95°C for 15:00 (min:sec), followed by 40 cycles of melting at 94°C for 00:30, annealing at 38°C, 01:00, elongation at 72°C, 01:00 and a final extension step at 72°C for 05:00.

#### E. faecium

Amplicons of *E. faecium* were generated using primer D8635: GAGCGGCCAAAGGGA GCAGAC (Akopyanz et al., 1992) with two different PCR conditions (Andrighetto et al., 2001): (i) 95°C for 15:00 (min:sec), followed by 35 cycles of melting at 94°C for 01:00, annealing at 47°C, 01:00, elongation at 72°C, 01:30 and a final extension step at 72°C for 10:00. (ii) Pre-PCR Cycle with 95°C, 15:00; 40°C, 05:00; 72°C, 05:00, followed by 35 cycles of 94°C, 01:00; 52°C, 01:00; 72°C, 02:00 and a final extension step at 72°C for 10:00.

### Investigation of the emeA-genotype

All enterococci with DDAC-MICs > 1.4 mg/l were previously shown to be negative for *qac*-genes (*qac* A/B, *smr* [qacC/qacD], *qac*EΔ1, *qac*G, *qac*J *qac*H/*qac*Z; Bischoff et al., 2012); one of the isolates was a *qac*A/B-negative variant of a strain which was previously shown to harbor *qac*A/B (Bischoff et al., 2012). Since *eme*A, a multidrug efflux pump with sequence homology to the staphylococcal *nor*A gene, is suspected to contribute to reduced disinfectant susceptibility, 44 *E. faecalis*-isolates (*E. faecalis* DSM 2570; eight study strains with MIC less or equal 1.05 and 35 study strains with MIC > 1.4) were investigated for their *eme*A-genotype using primers *eme*A-fw GACTCAACGAGTGTTTCAGCCAA and *eme*A-rv ACGATAAAAAGCCCGTTCCTA as suggested by NCBI Primer Blast. PCR-conditions were denaturation at 94°C, 05:00 (min:sec), followed by 30 cycles of melting at 94°C, annealing at 63°C, 01:00, elongation at 72°C, 01:00 and a final extension step at 72°C, 05:00.

#### Melting curve analysis

Amplicons were investigated by conventional melting curve analysis under the following conditions: step 1: 95°C, slope 20°C/sec; step 2: 65°C, hold-time 30 s., slope 20°C/sec; step 3: 95°C, slope 0.1°C/sec. Delta Tm was recorded as follows: Tm (wildtype) minus Tm (variant), with Tm = temperature at the maximum value for -d(F1)/dT (maximum of the melting peak). Peaks were suspected to be different if Delta Tm was at least 0.6°C. A diversity of peaks was further investigated by sequence analysis.

#### Sequence analysis

DSM-reference strain *E. faecalis* DSM 2570 (ATCC 29212), for which an *eme*A-sequence is already recorded in Genbank (AB091338.1 GI:22775586) and three *eme*A-amplicons of study isolates were externally sequenced (Sequiserve, Vaterstetten, Germany). Sequences were compared with each other and with nucleotide records—including nine *eme*A-sequences of *E. faecalis*—in Genbank using the default NCBI-BLAST-program for highly similar sequences.

#### Relative transcription of emeA-variants

The transcription of *eme*A in relation to the transcription of DNA coding for a partial sequence of the 23S-rRNA was investigated by RT-PCR as previously described (Schwaiger et al., 2012), using the *eme*A-primers as described above and primers GTAGTCCACAGCTTCGGTAATATGT and AACTAGGATGTTGGCTTAG AAGCA for a 23S-rDNA-fragment as described elsewhere (Hancock et al., 2003). For a preliminary impression, differences in gene transcription were calculated using the 2^∧^-DeltaDeltaCt-method.

### Statistics

The prevalence of DDAC-MICs > 1.4 mg/l within the groups of antibiotic resistant and antibiotic susceptible *E. faecalis* and *E. faecium* was assessed in a chi-squared test. If expected numbers per cell were below five, a Fisher's Exact test was applied. A non-parametric procedure (Mann–Whitney U) was used to compare the MIC-values of DDAC as well as the MIC-values of antibiotics in strains with different *eme*A-types.

## Results

### Susceptibility of *E. faecalis* and *E. faecium* to DDAC and antibiotics

#### MIC-values for DDAC

MIC-values for DDAC differed from wildtype-MIC-values only in a small fraction of *E. faecalis* or *E. faecium* (Table 2), and only in a very moderate way (Table 3). The highest prevalence of DDAC-MICs > 1.4 mg/l was seen in enterococci from milk and dairy products (14.5%), while the prevalence in livestock was generally low (0–4%). The maximum multiplication of MIC-values was 3.3-fold (1.05 vs. 3.5 mg/l).

**Table 2:** **Prevalence of *E. faecalis* and *E. faecium* with DDAC-MICs > 1.4 mg/l within antimicrobial-susceptible and –resistant isolates of different sources**.

**Source**	**Prevalence of strains with DDAC-MICs > 1.4 mg/l (n) within all investigated isolates (N)**							
	**Total**	**Total%**	**GNH^a^ susceptible**	**GNH resistant**	**SNH^b^ susceptible**	**SNH resistant**	**AMP^c^ susceptible**	**AMP resistant**
***E. faecalis***								
Hospital: human blood	6 (88)	6.8	6 (62)	0 (26)	5 (60)	1 (28)	6 (85)	0 (3)
Hospital: human feces	8 (210)	3.8	7 (144)	1 (56)	6 (177)	2 (33)	8 (210)	0 (0)
Outpatient feces	3 (102)	2.9	3 (94)	0 (8)	3 (96)	0 (16)	3 (102)	0 (0)
Swine, feces	0 (76)	0.0						
Cattle, feces	0 (50)	0.0						
Cattle, matistis milk	0 (50)	0.0						
Chicken, feces	2 (50)	4.0	2 (48)	0 (2)	2 (46)	0 (4)	2 (50)	0 (0)
Milk and dairy products	14 (96)	14.6	13 (95)	1^*^ (1)	14 (95)	0 (1)	2 (96)	0 (0)
Poultry	2 (54)	3.7	2 (54)	0 (0)	2 (43)	0 (11)	2 (54)	0 (0)
Beef	3 (30)	10.0	3 (30)	0 (0)	3 (29)	0 (1)	3 (30)	0 (0)
Pork	1 (28)	3.6	1 (26)	0 (2)	1 (25)	0 (3)	1 (28)	0 (0)
***E. faecium***								
Hospital: human blood	3 (45)	6.7	1 (31)	2 (14)	1 (32)	2 (13)	0 (7)	3 (38)
Hospital: human feces	2 (52)	3.8	0 (42)	2^*^ (10)	0 (43)	2^*^ (9)	0 (38)	2^*^ (14)
Outpatient: feces	2 (38)	5.3	2 (38)	0 (0)	2 (36)	0 (2)	2 (37)	0 (1)

a *GNH gentamicin (high level)*.

b *SNH streptomycin high level*.

c *AMP ampicillin*.

* *Differs significantly in a Fisher's Exact Test from the overall prevalence in this study, *p* < *0.05**.

Bold: emphasized for the sake of clarity

**Table 3:** **Minimal inhibitory concentrations and antimicrobial resistance of antiseptic-tolerant (MIC > 1.4 mg DDAC/l) *Enterococcus* isolates**.

**Species**	**Source**	***n***	**Range MIC DDAC (mg/l, 8-fold assay; 0.7 mg/l = 0.001%)**	**emeA-type**	*qac*-gene^*^	**Resistotype**
*E. faecalis*	Hospital: Human blood	1	2.1	1	–	CIP ENR LEV MOX DOX ERY TLS TEL SNH
		1^a^	2.1	2	–	DOX ERY
		1^a^	2.45–3.5	n.d.	qacA/B^+^^1^	DOX ERY
			1.75	2	qacA/B^−^	DOX ERY
		1	1.05–>1.4^**^	1	–	DOX
		2	2.1	0	–	–
	Human feces	1	1.75	0	–	CIP ENR LEV MOX ERY TLS TEL GNH SNH
		1	2.45–3.5	n.d.	–	DOX ERY SNH
		1	1.75	2	–	DOX ERY TLS
		1^b^	1.75	1	–	DOX ERY
		1	2.1	0	–	DOX
		1	1.75	0	–	–
		1^b^	2.8–3.5	2	–	–
		1^b^	3.5	1	–	–
	Outpatients: Human feces	1	2.1	2	–	DOX ERY
		1	2.1–2.45	2	–	–
		1	2.45–3.5	2	–	
	Animal: Chicken	2	1.05–1.75	1/0	–	–
	Milk and dairy products	1	1.75	1	–	ERY TLS TEL GNH
		2	2.1	0 2	–	ERY
		3	1.75	n.d. /1	–	–
		1	1.75–2.1	/0	–	
		1	2.1	x		
		5	2.1–2.45	x/1/2/0/0	–	
		1	2.1–3.5	2		–
		1		2	–	
	Meat: Poultry	1	1.05–>1.4^**^	0	–	–
		1	2.1	1	–	
	Beef	1	1.75	0	–	ERY
		1	1.75	2	–	–
		1	2.1	2	–	–
	Pork	1	2.1	2	–	ERY
*E. faecium*	Hospital: Human blood	1	1.75	n.d.	–	AMC AMP MZL IMP MER CIP ENR LEV MOX ERY TLS TEL GNH
		1^c^	1.75	n.d.	–	AMC AMP MZL IMP MER CIP ENR LEV MOX ERY TLS TEL SNH
		1^c^	1.75	n.d.	–	AMC AMP MZL IMP MER CIP ENR LEV MOX ERY TLS TEL SNH GNH
	Human feces	1^d^	1.75	n.d.	–	AMC AMP MZL IMP MER CIP ENR LEV MOX ERY TLS TEL SNH GNH
		1^d^	1.75	n.d.	–	AMC AMP MZL IMP MER CIP ENR LEV MOX ERY TLS TEL SNH GNH
	Outpatients: Human feces	1^e^	1.05–>1.4^**^	n.d.	–	ERY TLS LIZ
	Healthy humans:					
		1^e^	1.4–>1.4^**^	n.d.	–	CIP ENR ERY
		1	2.8	n.d.	n.d.	CIP ENR DOX GNH

1 *(Bischoff et al., 2012)*.

a,b,c,d Originating from different patients of the same hospital in the same period of investigation, respectively;

e *isolates of two different outpatients, sent in from the same laboratory in the same period of investigation*.

*DDAC, Didecyldimethylammoniumchloride; AMC, Amoxicillin + Clavulanate; AMP, Ampicillin; MZL, Mezlocillin; IMP, Imipenem; MER, Meropenem; CIP, Ciprofloxacin; ENR, Enrofloxacin; LEV, Levofloxacin; MOX, Moxifloxacin; ERY, Erythromycin; TLS, Tylosin; TEL, Telithromycin; GNH, Gentamicin High Level; SNH, Streptomycin High Level; LIZ, Linezolid*.

* *Investigated qac-genes: qacA/B, qacC, qacD, qacEΔ1, qacG, qacH/qacZ, qacJ*.

** *DDAC-tolerance was reproduced twice, but not at the third time*.

All “non-wildtype” MIC-values for DDAC (> 1.4 mg/l) were confirmed in the macrodilution test.

MBC-values did not exceed the MIC-values in any case (MBC = MIC in 44/44 isolates).

#### Adaptation to increasing DDAC-concentrations

**Figure 1:**
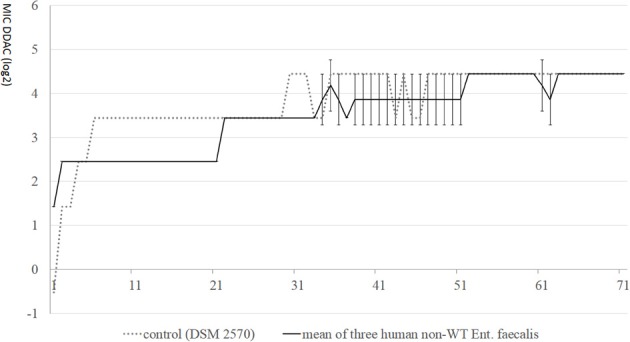
**MIC-values (log2) of didecyldimethylammoniumchloride (DDAC) in *E. faecalis* with initial MICs > 1.4 mg/l, compared with control DSM 2570 during 70 days of stepwise adaptation**.

All isolates which were used in the adaptation test were adapted to grow in higher concentrations (up to 21.9 mg/l) during 70 days of adaptation. Despite DDAC-MIC-values of 2.7 mg/l at the beginning, three human *E. faecalis* strains were not adapted to higher concentrations than the *E. faecalis* DSM 2570 strain with an initial DDAC-MIC-value of 0.7 mg/l (Figure 1): at day 5 of the adaptation experiment, DSM 2570 reached the same level as the non-wildtype strains. The DDAC-MIC-value for DSM 2570 increased further on day 6, while the non-wildtype strains had DDAC-MIC-values of 2.7 mg/l until day 22; all strains (including the DSM-strain) had DDAC-MICs of 21.9 mg/l (4.45 log2) on day 71.

#### MIC-values for antibiotics

The fraction of DDAC-MICs < 1.4 mg/l or > 1.4 mg/l within gentamicin/streptomycin-high-level- and ampicillin-resistant or -susceptible strains of *E. faecalis* and *E. faecium* is shown in Table 2. No significant differences were found for *E. faecalis*—neither for these substances, nor for any of the other investigated antibiotics (data not shown). However, for human hospital-derived *E. faecium* a Fisher's Exact Test revealed that DDAC-MICs > 1.4 mg/l *Enterococcus* were significantly (*p* < 0.05) more prevalent in streptomycin-high-level-resistant isolates: Of the clinical isolates, 1 out of 75 streptomycin-susceptible strains (1.3%) vs. 4 out of 22 streptomycin-resistant strains (18.2%) were inhibited by DDAC-concentrations > 1.4 mg/l. Significantly elevated prevalences of isolates with DDAC-MICs > 1.4 mg/l were also seen within clinical gentamicin- and ampicillin-resistant isolates (Table 2).

### Phylogenetic diversity of strains with DDAC-MIC > 1.4 mg/l

**Figure 2:**
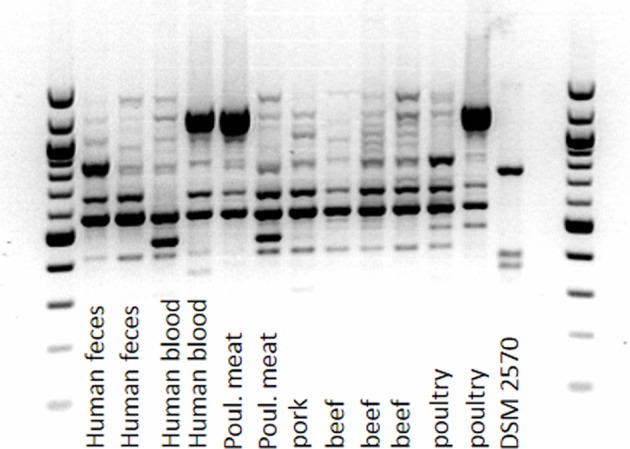
**Amplicon-typing (RAPD-PCR) of *E. faecalis* (MIC of DDAC > 1.4 mg/l) from different sources**.

In order to reveal whether enterococci with DDAC-MICs > 1.4 mg/l belonged to one or more clonal lineages, the phylogenetic diversity of the study isolates with DDAC_MIC > 1.4 mg/l was investigated by RAPD-PCR. The investigated strains of *E. faecalis* belonged to at least three distinct RAPD-types. However, compared to the completely distinct RAPD-profile of the reference strain, profiles of strains with DDAC-MIC > 1.4 mg/l were more similar, indicating a certain degree of phylogenetic relatedness, but no clonality (an example is given in Figure 2). One identical RAPD-profile was shared by one isolate from human blood, from poultry and from poultry meat, respectively (Figure 2).

**Figure 3:**
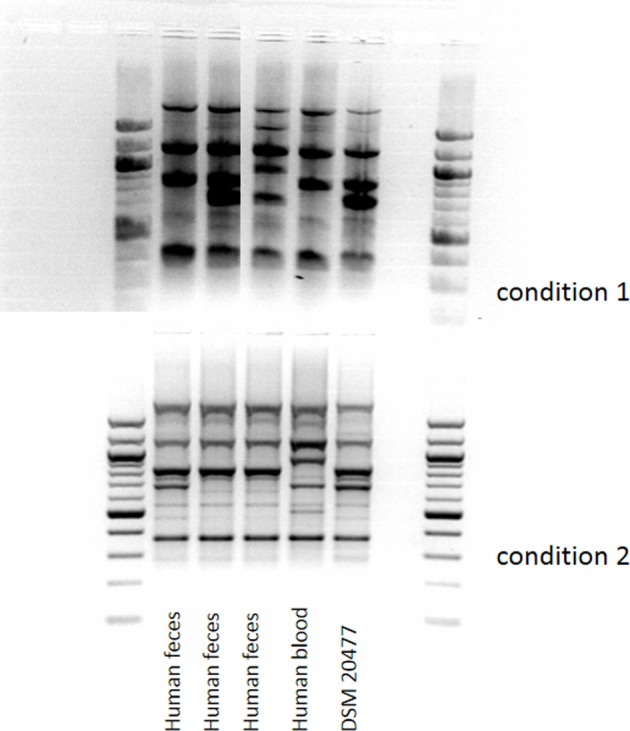
**Amplicon-typing (RAPD-PCR, two different conditions) of *E. faecalis* (MIC of DDAC > 1.4 mg/l) from different sources**.

The RAPD-profiles of *E. faecium* with DDAC-MICs > 1.4 mg/l were similar for the fecal isolates including the (wildtype) DSM type strain, but differed from the bloodstream isolate (Figure 3).

### emeA-genotype in *E. faecalis* with DDAC-MICs > 1.4 mg/l

#### Melting curve analysis

**Figure 4:**
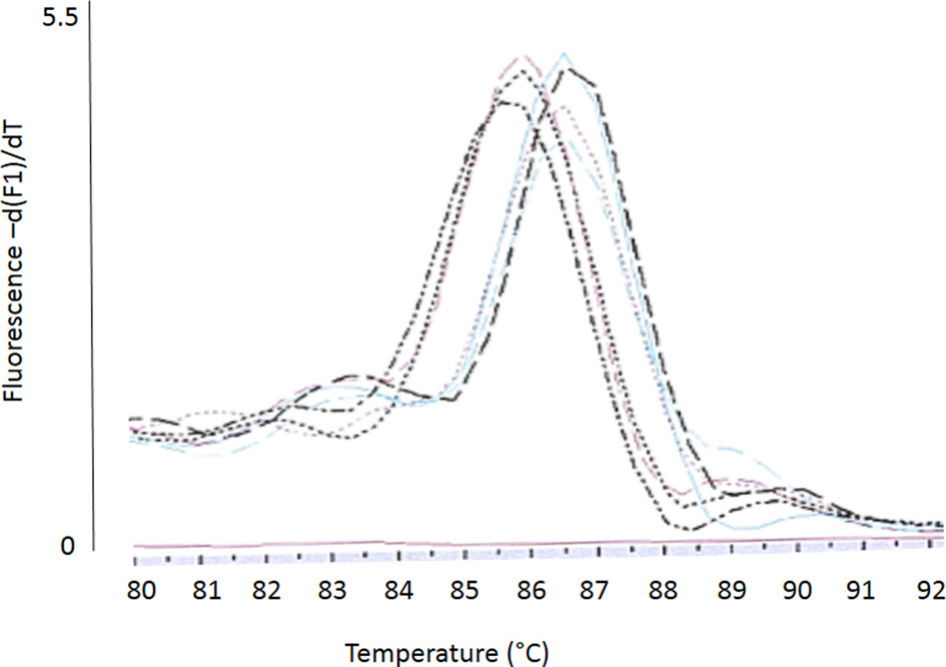
**Conventional melting curve analysis (Lightcycler I.I, SYBR-Green) of *eme*A-amplicons resulting in two different *eme*A types: type 1 (higher Tm) and type 2 (lower Tm)**.

Conventional melting curve analysis of *E. faecalis*-*eme*A-amplicons resulted in two clearly distinguishable peaks (type 1 and 2, Figure 4) at approximately 87and 86°C with Delta *Tm* = 0.9 ± 0.3°C). Of all investigated isolates, 12 could be clearly attributed to type 1 and 13 could be clearly attributed to type 2 (Table 3). The other isolates had a Delta Tm which could not be unambiguously assigned to one of both types (type 0) or had a Tm higher than type 1 (type x). *E. faecalis* DSM 2570 represented the prototype for the higher Tm-value (type 1). This strain and three prototypes for the lower Tm value (type 2) were externally sequenced. Results of a sequence comparison using NCBI-Primer-Blast are given in Table 4. The amplicon of DSM 2570 was completely identical to the corresponding sequence of DSM 2570 = ATCC 29212 recorded in Genbank by Lee et al. (2003) (Accession AB091338). The amplicons of the three sequenced study-strains differed from this sequence in 9 nucleotides; they matched two other recorded Genbank-sequences: *E. faecalis* D32 (Accession CP003726) and *E. faecalis* 62 (Accession CP002491).

**Table 4:** **Polymorphism analysis (NCBI BLAST) of *eme*A-amplicons as sequenced in the present study**.

**Source**	**MIC-value (mg/l)**	**Melting type**	**Polymorphic nucleotides**
Goulash (pork)	2.0	1	__141_G__251_G^*^__305_G__311_**T**__320_**T**__392_T__402_G__407_**T**__431_**T**__479_**A**__491_T__551_**T**__560_T__563_a__572_g__779_**T**__855_**T**_
Whipped cream	2.2	1	Sequence as above
Child (gastro-intestinal disorder)	2.7	1	Sequence as above
DSMZ (urine)	1.05	2	__141_G__251_A__305_G__311_C__320_G__392_T__402_G__407_G G__491_T__551_A__560_T__563_a__572_g__779_C__855_C___431_C__479_

*Bold letters: differ from DSM 2570. Other letters: identical with DSM 2570 but variable in other E. faecalis-emeA-entries in the Genbank database. _ spacer for conserved regions in all genbank entries*.

* *Unique variant not seen in any other genbank-entry*.

All differences between the DSM-strain and the sequenced study strains (as well as D32 and 62) were synonymous substitutions which do not affect the sequence of amino acids.

The median DDAC-MIC-value for investigated isolates with type 1 melting curves was 1.5 (1.05–3.5) mg/l, the median MIC for isolates with type 2 melting curves was 2.1 (1.75–3.2) mg/l, resulting in a significant difference in a non-parametric test (Mann–Whitney U, *p* < 0.01). Of eight investigated wildtype strains (MIC < 1.4 mg/l), none was attributed to melting type 2 (Table 3).

The number of phenotypic antibiotic resistances was 0–3 in isolates with *eme*A-type 2 and 0–9 in type 1 isolates. The median MIC-values of antibiotics differed only insignificantly between isolates of both melting types (Mann–Whitney U, *p* > 0.05).

#### Relative transcript level for two different emeA-variants

In relation to the basal transcription of DNA coding for a partial sequence of the 23 s rRNA, relative transcript levels for *eme*A-variants of type 2 were on average slightly higher than for *eme*A-variants of type 1 (mean 2^∧^-DeltaDeltaCt = 5.78). 2^∧^-DeltaDeltaCt-values (type 2 vs. type 1) ranged from 0.2 to 314.5. The maximum of 2^∧^-DeltaDeltaCt-values within type 1 was 116.4, the maximum of 2^∧^-DeltaDeltaCt-values within type 2 was 13.9, indicating a higher homogeneity in *eme*A-transcription within the type-2-group.

### Isolation of bacteria from disinfectant boot bath (active ingredient: 1.1% formic acid)

#### Bacterial identification

From boot bath disinfectant (1.1% formic acid), 42 bacterial strains were isolated and differentiated (Table 5). *E. coli* (*n* = 7) was only isolated from swabs substituted with inactivation medium. Other Enterobacteriaceae (*Providencia rettgerie*, *Acinetobacter lwoffi, Enterobacter cloacae*) were also isolated without this inactivation step. Gram-positive cocci (staphylococci and enterococci) were found in all kinds of sample including the disinfectant solution, although the latter was predominantly positive for *Bacillus* spp.

**Table 5:** **Antimicrobial resistance and susceptibility to disinfectants of bacteria isolated from boot bath disinfectant (2% concentrate = 1.1% formic acid)**.

**Source/Identity**	**MIC formic acid in %**	**MIC DDAC-stock solution (7 g/100 g) in %**	**Acquired resistance profile**	**Natural resistance profile**
**SWAB (AFTER INACTIVATION)**				
*Bacillus cereus* group	0.006	0.001	CIP FOS	CEC
*Enterococcus hirae*	0.003	0.008	CLI ERY ROX TYL DOX	MZL MER
*Staphylococcus saprophyticus*	0.07	0.004	TYL	–
*Escherichia coli* (*n* = 2)	0.14	0.008	–	GN^2^
*Escherichia coli*	0.14	0.008	DOX	GN
*Escherichia coli*	0.14	0.008	CEC	GN
*Escherichia coli* (*n* = 2)	0.14	0.008	DOX CEC	GN
*Escherichia coli*	0.14	0.008	AMC	GN
*Escherichia coli* ß-D-Gluc. neg (*n* = 2)	0.14	0.008	–	GN
*Citrobacter freundii*	0.14	0.008	–	GN + AMC CEC CEZ COX CXM
*Lactose-neg*. Enterobacteriaceae	n.d.	>0.01	CEC DOX ENR FOS	
**SWAB (NO INACTIVATION)**				
*Unidentified, double-zone hemolytic obligate anaerobe*	n.d.^1^	n.d.^1^	n.d.^1^	n.d.
*Unidentified Gram-negative*	n.d.	n.d.	GN + AMC CEC CEZ CXM CIP	
*Enterobacter cloacae*	0.14	0.03	FOS	GN + AMC CEC COX CEZ CXM
*Citrobacter* sp.	n.d.	>0.01	–	GN
*Citrobacter freundii*	0.14	0.008	–	GN + AMC CEC CEZ COX CXM
*Klebsiella oxytoca*	n.d.	>0.01	CEZ FOS	GN
*Acinetobacter lwoffi*	0.06	0.002	FOS	GN + AMC CEC CEZ COX CXM
*Providencia rettgerie*	0.14	0.03	AMC CEC CEZ FOS DOX TOB	GN
*Providencia rettgerie*	0.14	0.03	FOS DOX TOB	GN
*Moraxella* sp.	n.d.	>0.01	–	GN + AMC CEC CEZ COX CXM
**DISINFECTANT FLUID**				
*Bacillus cereus* group	0.003	0.001	–	AMC CEC CEZ COX CXM OXA PEN
*Bacillus licheniformis* (*n* = 3)	0.01	0.002–0.03	CLI ERY TEL FOS	CXM OXA PEN
*Bacillus subtilis* group	n.d.	>0.01	–	FOS
*Bacillus megaterium*	0.006	0.001	–	FOS CLI
*Bacillus pumilus*	0.003	0.001	–	–
*Clostridium perfringens*	0.003	0.008	n.d.	n.d.
*Enterococcus faecium* (*n* = 2)	0.02	0.008	CLI	ERT FOS MER MOX
*Enterococcus faecalis* (*n* = 2)	0.01	0.008	ERY TLS	CLI SYN
*Staphylococcus aureus*	0.003	0.008	–	–

1 *No growth in TS-broth, CAMHB, and Wilkens Chalgren broth in aerobic or anaerobic conditions*.

2 *GN: natural resistance to antibiotics which are ineffective in Gram-negatives like benzylpenicillin, macrolides, lincosamides, streptogramins. DDAC, Didecyldimethylammoniumchloride; AMC, Amoxicillin + clavulanate; AMP, Ampicillin; CEC, Cefaclor; CEZ, Cefazolin; CIP, Ciprofloxacin; COX, Cefoxitin; CXM, Cefuroxime; FOS, Fosfomycin; DOX, Doxycycline; MER, Meropenem; ENR, Enrofloxacin; ERT, Ertapenem; ERY, Erythromycin; LEV, Levofloxacin; MOX, Moxifloxacin; TLS, Tylosin; TEL, Telithromycin*.

#### MIC-values for formic acid and DDAC

MIC-values for the formic acid based disinfectant were 0.003–0.14% and thus below the used concentration of 1.1% formic acid, indicating that the isolated bacteria were not able to replicate in the disinfectant boot bath. In general, Gram-negative bacteria had higher MIC-values for formic acid (0.03–0.14%, median 0.14%) than Gram-positive bacteria (0.003–0.07%, median 0.01%). Besides one isolate, all strains had MIC-values comparable to a DSM reference strain of the same genus; one *S. saprophyticus* strain had a MIC of 0.07%, while *S. aureus* (DSM 1104) and one non-hemolytic *S. aureus* from the disinfectant fluid had MIC-values of 0.003% formic acid. MIC-values for DDAC ranged from 1.4 to 21.9 mg/l (0.002–0.03% concentrate, median: 0.008%) in Gram-negatives, with the highest values seen in *P. rettgerie* and *E. cloacae*, while *A. lwoffii* was the most susceptible Gram-negative strain. In Gram-positives, DDAC-MIC-values were 0.7–21.9 mg/l (0.001–0.03% concentrate, median: 0.008%), with the highest values seen in one of the three *B. licheniformis* isolates. *E. faecalis* and *E. faecium* isolates from disinfectant boot bath (active agent: formic acid) had non-wildtype MICs (0.008% or 5.6 mg/l) for DDAC.

#### Natural or acquired resistance to antibiotics

Of 42 strains isolated from disinfectant boot bath, 9 (21.4%, including *E. coli*) were resistant to amoxicillin + clavulanate, 5 (11.9%) to ciprofloxacin, 6 (14.3%) to doxycycline and 2 (4.8%) to tobramycin. *P. rettgerie* was multiresistant (amoxicillin + clavulanate, doxycycline, tobramycin). *Bacillus cereus* group isolates were intrinsically resistant to amoxicillin + clavulanate; other Bacillus isolates (*B. licheniformis*, *n* = 3, unidentified *Bacillus* sp.) were resistant to clindamycin, erythromycin and telithromycin, or to clindamycin only (*B. megaterium*). No antibiotic resistance was found in *B. pumilus* and *B. subtilis*.

## Discussion

Reports on a link of antibiotic resistance and reduced susceptibility to disinfectants are inconsistent: Russell concluded from his review that “bacteria showing reduced susceptibility to biocides may or may not be more resistant to antibiotics” (Russell, 2002). This might be due to species-specific differences, since many of the studies which report such a link are related to staphylococci (Zmantar et al., 2011). Since enterococci have rarely been representatively investigated up to now, this study aimed to provide completing data. The investigated disinfectant—a quaternary ammonium compound, DDAC—is used in every relevant context (food-producing environment, livestock, clinical settings) and listed by the German Veterinary Medical Society (DVG, 2008) as well as the German Association for Hygiene and Microbiology (DGHM), which are the most important reference institutions for recommending disinfectants in Germany. Recommended concentrations of the stock solution in the ready to use fluid range from 0.5 to 4% (350–2800 mg DDAC/l).

None of the investigated *Enterococcus* isolates—whether antibiotic resistant or not—was able to grow in a DDAC concentration recommended for *in praxi* use (0.5% = 5 ml stock solution per liter; stock solution = 7 g DDAC/100 g). However, since disinfectants are applied to frequently cleaned environments, rinsing might shape environmental niches with low concentrations of disinfectants. This is especially true in case of improper use (e.g., application to wet surface), but might also happen with proper use, since DDAC is non-volatile and stable to hydrolysis (Juergensen and Busnarda, 2000). Thus, wastewater disposal lines might provide permanent contact between bacteria and low concentrations of disinfectants. The same might be true for biofilm-associated bacteria in production lines like milklines. Relating to this, it might be interesting that the prevalence of DDAC-MICs > 1.4 mg/l was comparatively high in enterococci from dairy products.

The maximum observed MIC in our field study was 3.5 (0.005%), but after 70 days of adaptation in the laboratory, MICs of 21.9 mg/l (0.03%) were observed. Thus, permanent contact to low concentrations of disinfectants might considerably lower the susceptibility for these disinfectants. However, in the present study we could not substantiate that bacteria with higher initial MIC-values are more rapidly adapted to rising concentrations of QACs, as observed by others (Sidhu et al., 2002a,b).

With the macrodilution test followed by plating, we could not find differences between the minimum inhibitory and minimum bactericidal concentration of enterococci. Russell (2003) emphasizes that testing for lethal effects is superior to MIC-testing when assessing the effectiveness of disinfectants. This statement is underlined by the fact that Gram-negative bacteria are regularly reported to have higher MIC-values for disinfectants (Russell, 2001), suggesting lower susceptibility; however, at the same time, they might have lower MBC-values: Walsh et al. (2003) report that equal concentrations of a DDAC did more effectively reduce *E. coli* (but not *Pseudomonas* spp.) than *S. aureus*. Indeed, we could isolate Gram-positive cocci directly from 2% boot bath disinfectant fluid, while we did not find Gram-negatives in there.

In contrast to the expectation of higher disinfectant-MIC-values in Gram-negatives, in this study we found an identical maximum DDAC-MIC in Gram-positives (one of three *B. licheniformis*) and Gram-negatives (*E. cloacae*, *P. rettgerie*), as well as identical median values; only the minimum DDAC-MIC value was one log2-step lower in Gram-positives. However, MICs of formic acid were consistently higher in Gram-negatives.

Isolation of bacteria from disinfectant fluid does not necessarily mean that the disinfectant is ineffective, since the *in praxi* bacterial inoculum (and therefore the log10-reduction, for which several guidelines set the critical limit at greater than or equal to 5, e.g., BS EN 1276) was unknown. As long as bacteria do not reproduce, they are not enriched in the disinfectant fluid. Formic acid inhibited growth (=reproduction) of all bacteria isolated from boot bath disinfectant (1.1% formic acid) at concentrations = 0.14% in clean conditions. However, any disinfectant vanishes with time (or rinsing), and the protection of certain bacteria (while others are killed) will indeed affect the bacterial community in a post-disinfection environment (McBain et al., 2004). Thus it should be mentioned that we isolated *E. coli* with a derepressed AmpC-phenotype from the disinfectant boot bath, as well as multiresistant *P. rettgerie*. However, these isolates were accompanied by susceptible isolates of the same species (and thus ecological niche), and the *E. coli* were only found after an inactivation step.

Although all formic acid MIC-values were below the used concentration, one *S. saprophyticus* strain had a MIC-value of 0.07%, which was eye-catching compared to the *S. aureus* isolates with a MIC-value of 0.003%. Thus, high MIC-values might indeed be also indicative for an enhanced ability to survive in disinfectant surroundings (elevated MBC-values), even for structurally unrelated substances. However, vice versa low MIC-values do not exclude high MBC-values (Russell et al., 1999).

The presence of four *Enterococcus* isolates (two *E. faecalis*, two *E. faecium*) with non-wildtype MICs for DDAC is interesting, remembering the very low prevalence of such strains within these two species (e.g., *E. faecalis*: 0/50 porcine isolates) as seen in the representative DDAC-MIC-screening. The selection of such strains in a chemically completely distinct agent might be indicative that these enterococci use an unspecific way to reduce their susceptibility to chemical agents. Indeed, disinfectant resistance in enterococci has been linked to multidrug efflux pumps before (Poole, 2002). Since we investigated the *E. faecalis* isolates which showed DDAC-MICs > 1.4 mg/l in the screening nearly in vain for *qac*-genes (only four of 586 strains were positive, Bischoff et al., 2012), we had a closer look on the *eme*A-genotype, and noticed a variability of this *eme*A-genotype in a conventional melting curve analysis (verified by sequencing). However, strains with DDAC-MICs > 1.4 mg/l could not be consistently linked to one *eme*A-type, although the MIC-values of type 2 were statistically significantly higher than the MIC of type 1 and type 2 melting curves were absent in wildtype strains. The only difference between type 2 and the DSM-reference-strain (type 1) were nine synonymous substitutions. Thus, we do not assume that these substitutions affected the activity of the multidrug transporter. However, Berg and Martelius stated that an increase in synonymous substitutions might be caused by differences in gene expression (Berg and Martelius, 1995). Indeed, when we had a first glance at the mean relative transcript-levels of the two different *eme*A-variants, the relative transcript-levels for type 2 with the higher mean MIC-value was slightly (but insignificantly) higher. Further investigations on the gene expression level would be needed to verify this tendency, and further analysis of the promoter region might elucidate the cause for an enhanced transcription of *eme*A.

Multidrug efflux transporters of the *eme*A-type would only partially explain disinfectant-antibiotic “cross-resistance,” since they induce no or only minor changes in MIC-values for several antibiotics which were present in the multidrug resistant phenotypes of our isolates, like tetracyclines, macrolides or even fluoroquinolones (Lee et al., 2003). Interestingly, the number of phenotypic antibiotic resistances was 0–3 in isolates with *eme*A-type 2, but 0–9 in type 1 isolates, giving reason for ongoing investigations of a transferable nature of DDAC-non-wildtype phenotypes in the multiresistant isolate, due to another mechanism which is not related to *eme*A.

One multiresistant *E. faecalis* isolate mentioned above had a resistance profile similar to multiresistant *E. faecium* isolates (including gentamicin high level resistance and streptomycin high level resistance). For these *E. faecium* isolates, a statistically significant association of DDAC-MICs > 1.4 mg/l and gentamicin high level resistance, streptomycin high level resistance, and ampicillin resistance was seen. While *E. faecium* isolates (MIC DDAC > 1.4 mg/l) from the same source (human feces), but different institutions, had similar RAPD-types, the RAPD-type of an *E. faecium* from human bloodstream (MIC DDAC > 1.4 mg/l) differed substantially from the others. This indicates that the isolate was not clonally related to the other isolates and thus suggests another reason for congruent resistance phenotypes (like identical multiresistance plasmids). However, we could not prove the transferability of the phenotype or the presence of multiresistance plasmids up to now. It should also be mentioned that the “multiresistant + DDAC-tolerant”-phenotype was only found in few isolates from clinical settings, not in outpatients. However, the only gentamicin high level resistant *E. faecium* isolate which we found in healthy humans (1/32) also had a DDAC-MIC > 1.4 mg/l (data not shown).

## Conclusions

Resistance to *in-praxi*-concentrations of disinfectants still seems to be rare or even absent in livestock- or food-associated bacteria. This study gives some indication of a link between antibiotic resistance and (moderate) tolerance to disinfectants in a constrained number of isolates. Up to now the main driving force for the spread of such co- or cross-tolerant strains would be the use of antibiotics, not of disinfectants: selective advantage against disinfectants for the biocide-tolerant strains would be restrained to environmental niches (e.g., wastewater disposal lines), due to the fact that the moderately increased disinfectant-MIC-values were still far below disinfectant concentrations used *in praxi*. Thus, proper use of disinfectants in livestock surroundings still should be highly encouraged, as long as it is accompanied by general good hygiene praxis.

### Conflict of interest statement

The authors declare that the research was conducted in the absence of any commercial or financial relationships that could be construed as a potential conflict of interest.
